# Investigating the *ACE2* polymorphisms in COVID‐19 susceptibility: An *in silico* analysis

**DOI:** 10.1002/mgg3.1672

**Published:** 2021-04-05

**Authors:** Nasser Pouladi, Sepehr Abdolahi

**Affiliations:** ^1^ Department of Biology Faculty of Basic Science Azarbaijan Shahid Madani University Tabriz Iran; ^2^ Department of Molecular Biology and Cancer Research Azarbaijan Shahid Madani University Tabriz Iran; ^3^ Department of Biology Faculty of Basic Science Azarbaijan Shahid Madani University Tabriz Iran

**Keywords:** *ACE2* gene, COVID‐19, genetic susceptibility, polymorphisms, SARS‐CoV‐2

## Abstract

**Background:**

Novel coronavirus (SARS‐CoV‐2) became an epidemic disease and lead to a pneumonia outbreak first in December 2019 in Wuhan, China. The symptoms related to coronavirus disease‐19 (COVID‐19) were different ranging from mild to severe lung injury and multi‐organ failure symptoms, eventually leading to death, especially in older patients with other co‐morbidities. The receptor of this virus in the human cell is angiotensin‐converting enzyme 2 (ACE2).

**Methods:**

In this paper, we aimed to perform an *in silico* analysis of the frequently studied variants of the *ACE2* gene and determine the effects of the variants in mRNA secondary structure and binding affinity of cellular factors. Fourteen single‐nucleotide polymorphisms were selected based on previous studies and investigated.

**Results:**

All of the variants were analyzed in the RNAsnp database and three revealed a significant *p*‐value. The spliceAid2 database prediction showed that 7 out of 14 SNPs caused an alteration in a way that only the wild or mutated form was able to bind to proteins. The latter database also reported that three SNPs produces a dual form in which different specific proteins can bind to the sequence in a specific form (either wild or mutated form).

**Conclusion:**

Altogether, these estimations revealed the potential of variants in manipulation of the final stable form of ACE2 that can lead to different COVID‐19 susceptibility.

## Graphical abstract

In this paper, we aimed to perform an in silico analysis of the frequently studied variants of the ACE2 gene to determine the effects of the variants in mRNA secondary structure and binding affinity of splicing factors.

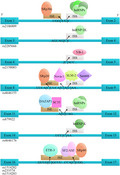

## 1 INTRODUCTION

The severe acute respiratory syndrome coronavirus (SARS‐CoV) was first emerged 17 years ago (Drosten et al., 2003). Later in December 2019 in Wuhan, China, a novel coronavirus (SARS‐CoV‐2) appeared and was found that it is able to infect humans (Gorbalenya et al., 2020) and lead to a pneumonia outbreak (Jiang et al., 2020). This virus causes coronavirus disease‐19 (COVID‐19) with mild to severe lung injury and multi‐organ failure symptoms, eventually leading to death, especially in older patients with other co‐morbidities. However, genetic or environmental factors increasing the susceptibility to SARS‐CoV‐2 remained unclear.

Angiotensin‐converting enzyme 2 (*ACE2*; OMIM accession number = 300335) was identified as the functional SARS‐CoV receptor *in vitro* and *in vivo* (Imai et al., 2005; Monteil et al., 2020). Also, *ACE2* gene contains 18 exons, and spans approximately 40 kb of genomic DNA on the human X‐chromosome and it is a homolog of ACE1 and exhibits 40% identity of amino acid sequence to its N‐ and C‐terminal domains (Harmer et al., 2002; Tipnis et al., 2000). Disease severity in mice infected with SARS‐CoV induced by human *ACE2* overexpression revealed that ACE2‐dependent viral entry into cells is a critical step (Kuba et al., 2005; Li et al., 2003; Yang et al., 2007). The main function of ACE2 in the body is blood pressure regulation through the renin–angiotensin system (Donoghue et al., 2000). ACE2 is a metalloprotease from renin–angiotensin system that is able to cleave angiotensin II into angiotensin 1–7 and angiotensin I into angiotensin 1–9 that is rapidly converted to angiotensin 1–7 by ACE1 (Tipnis et al., 2000). Susceptibility to SARS‐CoV was positively related to the ACE2 expression level in lung cells, as well as nine diverse cell lines (Hofmann et al., 2004; Jia et al., 2005). The primarily expression site of ACE2 in a normal adult human lung is alveolar epithelial type II cells (Guo et al., 2020). These cells produce surfactant which reduces lung surface tension, hence impeding alveoli from collapsing, therefore are crucial for the proper gas exchange function of the lungs (Dobbs, 1989).

Some specific genetic variations in the sequence of *ACE2* may affect the cell‐entry efficiency of viruses, either by changing its expression levels or causing higher binding affinity for SARS‐CoV‐2. Since ACE2 enzyme was discovered, researchers have explored the association of single‐nucleotide polymorphisms (SNPs) of the gene (localized on chromosome Xp22.2) with hypertension and other related heart diseases, with special attention to 14 single‐nucleotide polymorphisms including rs2285666, rs1978124, rs2074192, rs2106809, rs4830542, rs4240157, rs879922, rs2158083, rs233574, rs1514282, rs1514283, rs4646155, rs4646176, and rs4646188 (Fan et al., 2019; Lu et al., 2012; Luo et al., 2019; Niu et al., 2007; Pinheiro et al., 2019).

Despite all the efforts done by researchers, still there are no considerable studies focused on the effect of *ACE2* variants on different population's susceptibility to COVID‐19. In this paper, we aimed to perform an *in silico* analysis of these frequently studied variants of the *ACE2* gene to determine the effects of these variants on mRNA secondary structure and estimated a possible alteration in binding affinity of cellular factors due to the polymorphisms change. Besides, we aimed to bring the attention of scientific community to potential risk factor polymorphisms of the *ACE2* that should be investigated further.

## 2 MATERIALS AND METHODS

Data and the FASTA format of the *ACE2* (Genbank reference sequence and version number: NC_000023.**11**) sequence were gathered from the National Center for Biotechnology Information (NCBI) (https://www.ncbi.nlm.nih.gov/gene/59272) and used for further computational analysis.

The information on *ACE2* SNPs [reference SNP (rs) ID number, minor allele frequency, and variant type] was obtained from the NCBI dbSNP database (https://www.ncbi.nlm.nih.gov/snp).

SpliceAid2, a tool that allows us to know which proteins can bind to the desired RNA sequence, was used to investigate the effects of studied SNPs in protein binding affinity. This tool is entirely based on the human true splicing site and experimentally assessed target motif. The results of mutation effects on splicing binding in spliceAid2 database demonstrate information on creation of splice sites or strengthening of cryptic splice sites and provide information on intron retention, appearance, and disappearance of new alternative splice site forms (Piva et al., 2012).

RNAsnp tool has been developed to aid the prediction of SNP‐induced structural changes in local regions of the RNA secondary structure. This web server can predict local structure changes and report the exact location of the disrupted region and the significance of the structural change in the form of an empirical *p*‐value. Also, the web server can predict the structural effect of natural variants and to screen putative structure‐disruptive nucleotide variants for mutagenesis experiments (Sabarinathan et al., 2013).

## 3 RESULTS

**FIGURE 1:**
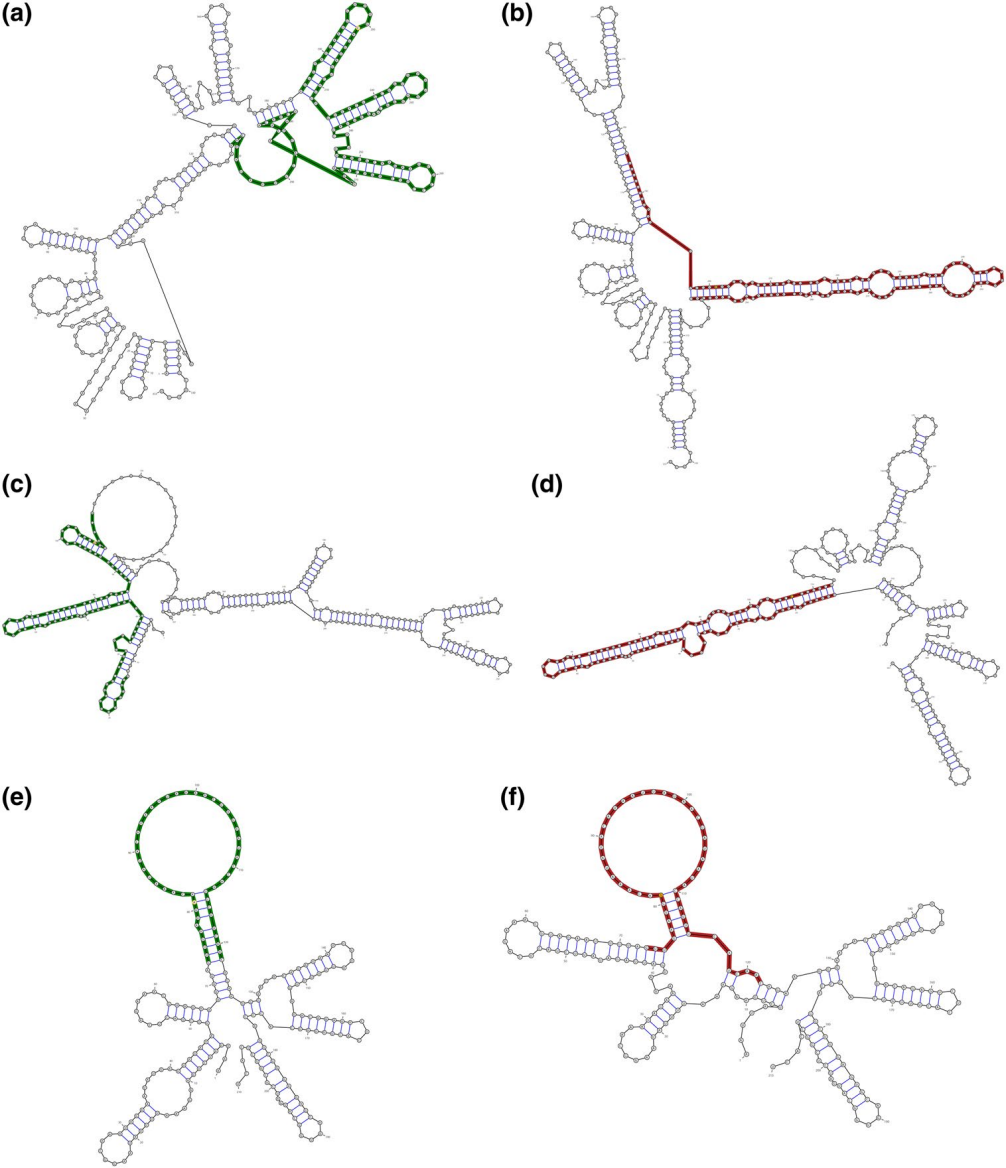
The effects of SNPs on secondary RNA structure by RNAsnp estimation. (a) Represents rs233574 in the wild type form wild type form when allele T exists, (b) shows a structural change in the wild type form when C allele substitutes. (c) Represents rs2074192 in the wild type form when allele T exists, (d) shows a structural change in the wild type form when C allele substitutes. (e) Represents rs4646188 in the wild type form when allele A exists, (f) shows a structural change in the wild type form when G allele substitutes

A total number of 14 SNPs of *ACE2* were selected and analyzed. All of the variants were subjected to RNAsnp database and results showed that three of them including rs233574, rs2074192, and rs4646188 had a significant p‐value (Table 1). These estimations predicted that these three SNPs may lead to a considerable RNA secondary structure changes (Figure 1).

**TABLE 1:** The *p*‐values of SNPs from RNAsnp server. A cut‐off value of 0.2 assigned by the server which establish *p*‐value greater than 0.2 as not significant value

Variant ID	*p*‐value*
rs4830542	0.922
rs233574	0.103
rs1514283	0.468
rs1514282	0.312
rs2074192	0.105
rs4240157	0.983
rs4646176	0.405
rs879922	0.305
rs4646155	0.977
rs4646188	0.186
rs2158083	0.965
rs2285666	0.618
rs2106809	0.646
rs1978124	0.996

* *p* < 0.20 consider as statistical significance.

**FIGURE 2:**
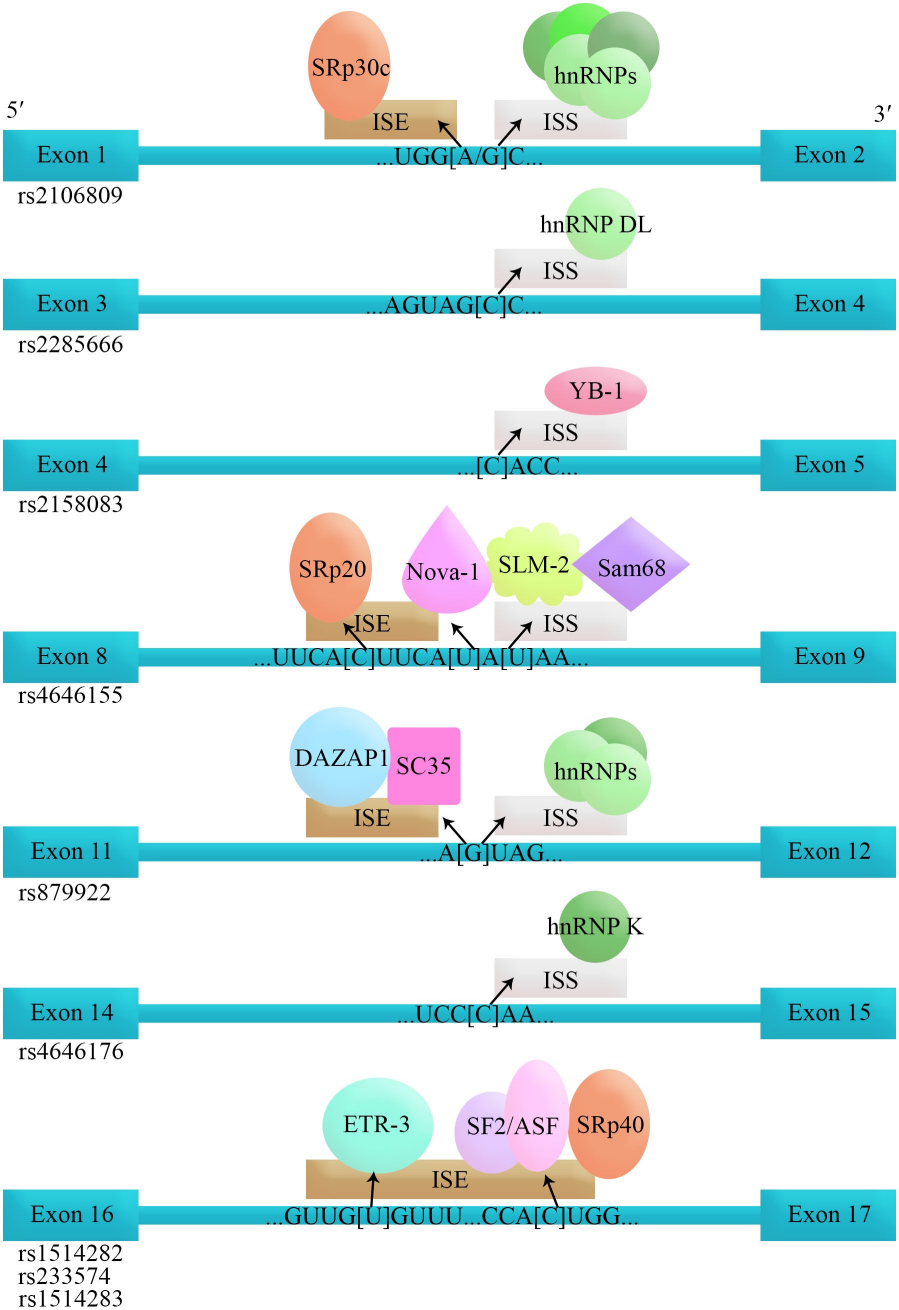
The schematic sketch of splicing factors binding to their relative sequence. Only the binding alleles are shown. The arrows show the creation of differential splicing regions and the corresponding splicing factors by the specific allele. Square brackets are demonstrating the polymorphism. ISE, intron splicing enhancer; ISS, intron splicing silencer

The selected variants were also subjected to the spliceAid2 website (Table 2, Figure 2). The database predicted that 5 out of 14 SNPs cause an alteration in the *ACE2* gene in a way that only the wild type form can bind to proteins. Two out of 14 induce changes that only the mutated form can bind to proteins. The other two SNPs produce dual form in which specific proteins bind to either wild or mutated sequence.

**TABLE 2:** SpliceAid2 server estimations

Variant ID	Wild type form (score)	Mutated type form (score)
rs4830542	—	—
rs233574	ETR‐3 (5)	
rs1514283		SF2/ASF (5); SRp40 (5)
rs1514282	ETR‐3 (5)	
rs2074192	—	—
rs4240157	—	—
rs4646176	hnRNP K (−5)	
rs879922		SC35 (5), DAZAP1 (5), hnRNP A0 (−5), hnRNP A1 (−5), hnRNP A2/B1 (−5), hnRNP D(−5), hnRNP DL (−5)
rs4646155	SRp20 (5)	Nova−1(5), SLM−2 (−5), Sam68 (−5)
rs4646188	—	—
rs2158083	YB−1 (5)	
rs2285666	hnRNP DL (−5)	
rs2106809	SRp30c (5)	hnRNP H1(−5), hnRNP H2(−5), hnRNP F (−5), hnRNP H3 (−5)
rs1978124	—	—

According to the scores provided by the server which can vary between −10 and 10: a positive score assigned to a sequence that facilitates exon definition which can be exonic splicing enhancer (ESE) or intronic splicing silencer (ISS). Relatively, a negative score assigned to a sequence that facilitates intron definition which can be exonic splicing silencer (ESS) or intronic splicing enhancer (ISE).

The rs233574 showed the secondary RNA change upon nucleotide alteration and demonstrated a splicing sequence creation in its wild type form. Hence, due to its importance based on its deleterious effect determined by both of the estimation tools, the allele frequency of this SNP in different populations (based on 1000 genome project) was drawn (Table 3).

**TABLE 3:**
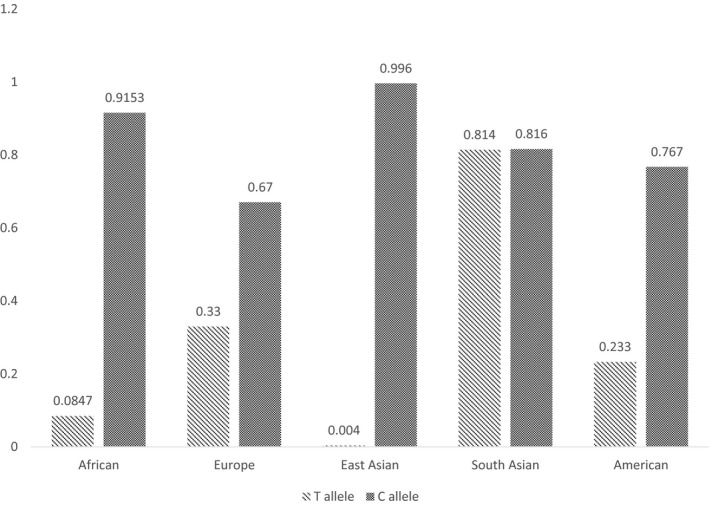
Allele frequency of the rs233574 polymorphism in different populations

## 4 DISCUSSION AND CONCLUSION

Human pathogenic SARS‐CoV‐2 enters cells through binding to *ACE2* expressed by epithelial cells of the lung and other organs (Wan et al., 2020). Patients with diabetes injected with ACE inhibitors showed a considerable overexpression of *ACE2* (Wan et al., 2020). One treatment for hypertension is ACE inhibitors, thus it is expected to upregulate the expression of *ACE2* in the patients (X. C. Li et al., 2017). *ACE2* can also be elevated by thiazolidinediones and ibuprofen. The urgent need for developing an efficient drug led to produce a human recombinant soluble ACE2 (hrsACE2) to inhibit the binding of SARS‐CoV‐2 to its receptor, ACE2. A study demonstrated that hrsACE2 can block the early entry of SARS‐CoV‐2 infections in host cells (Monteil et al., 2020). These data suggest that *ACE2* expression is increased in diabetes and treatment with ACE inhibitors increase *ACE2* expression. Consequently, the increased expression of *ACE2* can facilitate the infection with COVID‐19. Therefore Lei Fang et.al hypothesized that diabetes and hypertension treatment with ACE2‐stimulating drugs increases the risk of developing severe and fatal COVID‐19 (Fang et al., 2020).

Due to the role of ACE2 inhibitors in treating diseases including hypertension, cancer, and diabetes, the mentioned hypothesis could challenge the treatment method by these inhibitors (Monteil et al., 2020). A further feature to be investigated is the genetic predisposition for an increased risk of SARS‐CoV‐2 infection might be the *ACE2* polymorphisms that have been associated with the diseases. A combination of both *ACE2* polymorphism and therapy may define the sensitivity of a patient and thus requires careful considerations regarding determining the polymorphisms and choosing the best treatment option.

Until now, the role of *ACE2* polymorphisms has not been ruled out regarding its implications on COVID‐19 treatment. Thus, this study provided a computational analysis on the most important *ACE2* polymorphisms, to provide a firm ground for more investigations.

The results of this study showed that polymorphisms including rs233574, rs2074192, and rs4646188 with the global minor allele frequency (MAF) of 0.158, 0.363, and 0.061, respectively, were able to induce a significant RNA secondary structure change. These alterations may lead to dysregulations in *ACE2* transcription/translation or its protein stability, which in turn may result in changing COVID‐19 binding to ACE2 receptor and modulating SARS‐CoV‐2 pathogenesis. The secondary structure of mRNA induced by the mentioned polymorphisms may result in a protein to be more prone to proteases by exposing the sensitive amino acid sequence or by manipulating its proper folding.

ETR‐3, a member of the CELF family, regulates splicing by direct binding to the pre‐mRNA, binds U/G motifs, and directly activates exon inclusion *in vitro*. Binding and activation by ETR‐3 are directly antagonized by polypyrimidine tract‐binding protein (PTB) (Charlet et al., 2002; Faustino & Cooper, 2005). Occurrence of wild type allele of rs233574 and rs1514282 in the region results in binding of ETR‐3 protein and may induce exon inclusion in ACE2 gene. Otherwise, in the case of the mutated type allele of rs233574 and rs1514282, no specific protein binds. So, inducing an analogous ETR‐3 protein without exon inclusion activity may contribute to lower risk of infection in patients with the wild type form of rs233574.

The same role stands for the wild type form of rs2158083 and rs2285666, which lead to binding of YB‐1 and hnRNP DL, respectively. No specific protein binds in the mutated type form, resulting in exon retention. The human Y box‐binding protein‐1 (YB‐1) is a deoxyribonucleic acid (DNA)/ribonucleic acid (RNA)‐binding protein with pleiotropic functions. Several recent studies have indicated that YB‐1 is a spliceosome‐associated protein involved in alternative splicing as splicing enhancers (Wei et al., 2012). Heterogeneous ribonucleoprotein D‐like (hnRNPDL) is an RNA‐processing prion‐like protein with three alternative splicing (AS) isoforms, which lack none, one, or both of its two disordered domains. It has been suggested that alternative splicing might regulate the assembly properties of RNA‐processing proteins by controlling the incorporation of multivalent disordered regions in the isoforms. This, in turn, would modulate their activity in the downstream splicing program (Batlle et al., 2020). Thus, our results demonstrated that binding of YB‐1 and hnRNP DL to rs2158083 and rs2285666 would enhance the splicing and produce an ACE2 with more binding affinity to the SARS‐CoV‐2. Hence, finding therapeutic interventions to interact with YB‐1 and hnRNP DL on the *ACE2* would contribute to the lower susceptibility to COVID‐19.

Despite the binding of hnRNP K to the wild type form of rs4646176, this interaction causes an exon exclusion. HRNP K splicing factor binds to exonic or intronic sites and acts as either an activator or repressor of exon inclusion, exhibits a binding preference for CA‐rich elements (Liu et al., 1998). Our results confirmed the role of exonic inclusion of hnRNP K by the spliceAid2 estimation server.

Two of the studied SNPs showed a binding affinity to splicing factors only in their mutated type form, including rs1514283 and rs879922. In the case of rs1514283, SF2/ASF and SRp40 bind and lead to the creation of a new intron splicing enhancer (ISE) and exon inclusion. In the case of rs879922, SC35 and DAZAP1 bind, which may lead to exon inclusion. In addition, hnRNP A1, A0, A2/B1, D, and DL family bind to rs879922 sequence and create a new intronic splice silencer (ISS) and intron exclusion. The RNA affinity purification has identified hnRNP A1, A0, A2/B1, and D family as binding partners for ISSs (Choudhury et al., 2014).

SF2/ASF plays a role in preventing exon skipping, ensuring the accuracy of splicing, and regulating alternative splicing (Smith et al., 2006). DAZAP1 has been identified as a binding protein for several intronic splicing enhancers or ISSs in human cells (Choudhury et al., 2014). The last splicing factor, SC35, is necessary for the splicing of pre‐mRNA. (Caputi & Zahler, 2002).

The results of the SpliceAid2 server showed that the last two polymorphisms including rs4646155 and rs2106809 may bind to different specific splicing factors in both wild and mutated forms. The mentioned splicing factors are SRp20 and SRp30c and their binding results in exon retention.

The protein encoded by the SRp30c gene is a member of the serine/arginine (SR)‐rich family of pre‐mRNA splicing factors, which constitute part of the spliceosome. Each of these factors contains an RNA recognition motif (RRM) for binding RNA, and an RS domain for binding other proteins (Paradis et al., 2007). SRp20 (also called SRSF3) is the smallest member of the same family and regulates the splicing of numerous genes. It affects alternative splicing by interacting with RNA cis‐elements in a concentration‐ and cell differentiation‐dependent fashion (Corbo et al., 2013).

In the case of the mutated form of rs4646155, NOVA‐1 can bind to the region and induce an exon inclusion, on the other hand, SLM‐2 and Sam68 lead to ISS creation thus intron exclusion. *NOVA1* gene encodes a neuron‐specific RNA‐binding protein, a member of the NOVA family of paraneoplastic disease antigens, which is recognized and inhibited by paraneoplastic antibodies. These antibodies are found in the sera of patients with small‐cell lung cancer, breast cancer, and paraneoplastic opsoclonus‐ataxia (Beuth et al., 2007).

The protein that creates a new ISS, Sam68 (Src‐associated in mitosis 68 kDa), is the prototypic member of the STAR (Signal Transduction and Activation of RNA) family of RNA‐binding proteins, which regulate splicing in response to signaling cascades (Rajan et al., 2009; Sánchez‐Jiménez & Sánchez‐Margalet, 2013).

Furthermore, the mutated form of rs2106809 was able to bind to the hnRNP H protein family, which leads to a new ISS and intron exclusion (Caputi & Zahler, 2001). Our results revealed the function of rs4646155 and rs2106809 as two most imported SNPs in COVID‐19 susceptibility based on the spliceAid2 estimations. These two SNPs would bind to specific splicing factors, both in wild type and alternative forms, and result in exon inclusion and intron exclusion. Our *in silico* estimations suggest that all the investigated polymorphisms tend to increase the *ACE2* expression levels by affecting the splicing in the form of exon retention followed by intron exclusion. However, functional studies need to confirm these estimations.

Finally, the comparison of rs233574 frequency between different populations revealed that all populations, except for South Asian, have a more prevalent C allele. According to the ability of the rs233574 sequence to bind to the ETR‐3 splicing factor, in the presence of the T allele, and the fact that distribution of rs233574 in Asians is significantly different compared with other populations (Paniri et al., 2021), it can be concluded that COVID‐19 susceptibility may increase in South Asian population (WHO.int, 2020). In conclusion, regarding the results from two *in silico* servers, 3 out of 14 selected SNPs estimated to be able to alter the RNA secondary structure of *ACE2*. Furthermore, 9 out of 14 analyzed polymorphisms suggested to affect the splicing factor binding affinity. Altogether, these estimations revealed a potential role of the selected variants in the manipulation of the final stable form of ACE2 protein, which can lead to different COVID‐19 susceptibility. Thus, further investigations, especially case–control studies in *ACE2* polymorphisms, would shed light into COVID‐19 pandemic situation and may assist the researchers and clinicians in finding specific, suitable and efficient therapies.

## CONFLICT OF INTEREST

The authors declare that there is no conflict of interest and no fund was available.

## AUTHORS’ CONTRIBUTION

SA performed supervision, conceptualization, formal analysis, data curation, methodology, writing‐original draft preparation. NP did project administration, validation, visualization, writing‐review & editing.

## Data Availability

The data that support the findings of this study are openly available in Google Drive at here.
